# Primary Squamous Cell Carcinoma, a Rare Pathological Report of Pancreatic Cancer

**DOI:** 10.22088/cjim.12.0.407

**Published:** 2021

**Authors:** Pegah Farokhi, Alireza Sadeghi, Azadeh Moghaddas, Mitra Heidarpour, Saman Dinari

**Affiliations:** 1 Department of Clinical Pharmacy, Isfahan University of Medical Sciences, Isfahan, Iran; 2 Department of Internal Medicine, Faculty of Medicine, Isfahan University of Medical Sciences, Isfahan, Iran; 3 Department of Clinical Pharmacy, Faculty of Pharmacy, Isfahan University of Medical Sciences, Isfahan, Iran; 4 Department of Pathology, Faculty of Medicine, Isfahan University of Medical Sciences, Isfahan, Iran

**Keywords:** Primary squamous cell, Carcinoma, Pancreatic cancer, Survival, Chemotherapy

## Abstract

**Background:**

Primary squamous cell carcinoma (SCC) of the pancreas is a rare tumor and associated with poor prognosis. The diagnosis and optimal management of patients is still a matter of debate and not well-defined. Limited chemotherapy protocols, radiotherapy and surgical resection of the tumor were proposed for the management of patients suffering from SCC of the pancreas.

**Case Presentation:**

In this report, we introduced a 57-year-old man who was diagnosed with SCC of the pancreas along with liver metastasis. The patient underwent surgical resection and several adjuvant systemic chemotherapies including fluorouracil and taxane based regimens which were led to the 13- month overall survival.

**Conclusion:**

Although, the patients died from underlying tumor, the survival time before death was one of the longest time/period reported.

Pancreatic cancer, generally refers to a ductal adenocarcinoma of the pancreas, is categorized as a highly aggressive tumor and estimated to become the second leading cause of cancer-related deaths by 2030 (1). Exocrine elements are responsible for more than 95 percent of malignant neoplasms of the pancreas while adenocarcinoma represented approximately 85 percent of all pancreatic neoplasms. Of the several subtypes of ductal adenocarcinoma, mostly have a similar poor long-term prognosis. The more distinct term "exocrine pancreatic neoplasms" compose all tumors that are caused by the pancreatic ductal, acinar cells and their stem cells (2). Squamous cell carcinoma (SCC) is a subtype of ductal carcinoma, a non-endocrine part. It is one of the rare subtypes of pancreatic malignancy that correlates with poor prognosis. It is accounting for 0.5-2% of all malignancy of the pancreas (3). The diagnosis and optimal management of SCC of pancreas remains poorly defined. There is no available standard treatment protocol or guidelines for the management of SCC of pancreas mainly due to the rarity of the disease. Surgical resection, chemotherapy, and radiation were proposed as therapeutic approaches (2). Herein, a case of advanced SCC of the pancreatic with liver metastasis was reported. The patient underwent surgical resection followed by adjuvant chemotherapy by fluorouracil and taxane-based regimens. 

## Case presentation

A 57yearold man, with a known case of hypertension, was admitted at the emergency room of Milad hospital, Isfahan, Iran, in June 2018, with chief complaints of right abdominal pain (epigastric pain radiating to back and shoulder), vomiting, weight loss (more than 10 kg over the last 6 months) and weakness.

He had no history of smoking or alcohol consumption. The primary physical examination had tenderness in his left upper abdomen and paleness in the face. Initial investigations at the emergency room revealed that his baseline serum amylase (15.59 IU/l), lipase (17.16 U/l) and other routine laboratory test were within normal limits. However, the serum levels of carbohydrate antigen (CA) 19–9 (112.7IU/mL, reference: 0–37IU/mL) and CA-125 (239.3IU/mL, reference: 0–40IU/mL) were elevated. 

**Figure 1:**
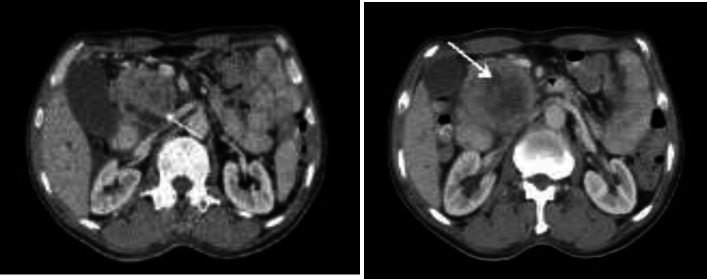
Computed tomography image showing an 8.8 x1.6.8 x1 cm mass in the head of pancreas

An upper gastrointestinal endoscopy had no major finding except an ignorable mucosal swelling. However, in ultrasonography investigation, hypoechoic and heterogeneous mass with size 66*71 cm in the head of the pancreas, and porta hepatitis mass suspicious to lymphadenopathy involvement have been detected. Abdominal and pelvic computed tomography (CT) has been considered for confirming the diagnosis and heterogeneous enhancing mass lesion measured 8.8x1.6.8.x1 cm in the pancreas’s head (figure 1) was reported accordingly. In addition, the mass displaced superior mesenteric vein (SMV), dilated the pancreatic duct and intra and extrahepatic bile duct (common bile duct was 20 mm in diameter). 

For further evaluation, patient was sent to oncology clinic of Omid Hospital, Isfahan, Iran affiliated to Isfahan University of Medical Sciences, specialized for the treatment of cancer patients. At first, the patient referred to surgical oncologist and due to tumor confirmation, the surgeon decided to perform tumor resection and tumor biopsy gathering during open pancreatomy surgery. Adenosquamous carcinoma, pancreatoblastoma and metastatic SCC from another primary site were contemplated as differential diagnosis. 

**Figure 2:**
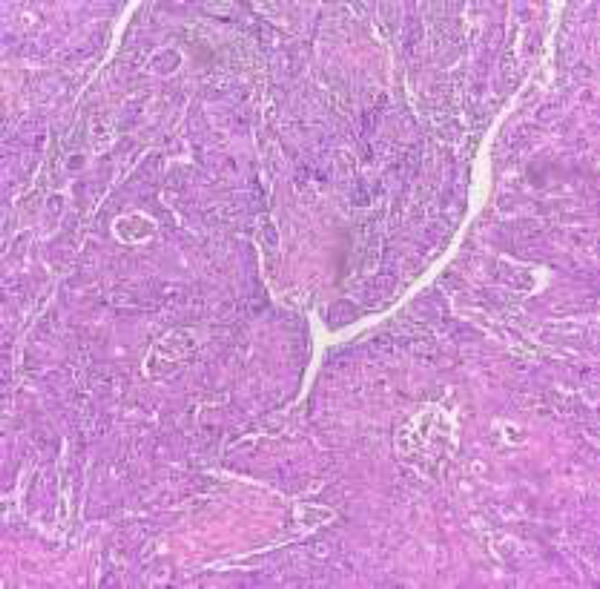
Markedly pleomorphic tumor cells in diffuse sheets showed clouds of squamous cells with keratin pearls displaying in the hemorrhagic necrotic tissue (hematoxylin and eosin staining, magnification, x200)

**Figure 3:**
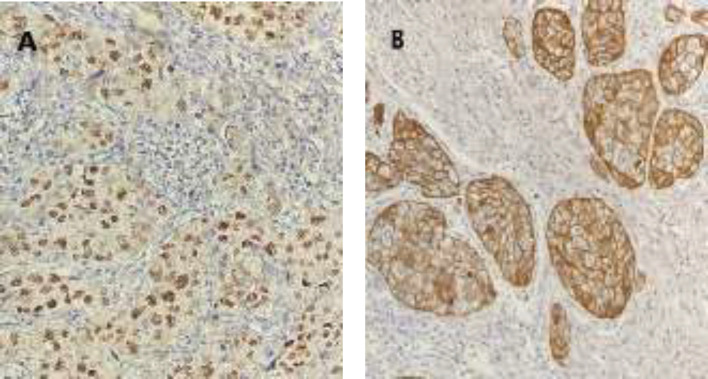
Histological finding showed a positive result for immunohistochemistry staining A, P63 marker B, CK5/6 marker (magnification, x400)

After surgery, the resected sample from the operating room including part of gastric and intestine tissue in the length of 36 cm and pancreas tissue in the 7*8*9 mm dimension were sent for histopathological evaluation. In microscopic examination, there was evidence of tumor cells with high nucleus- cytoplasm ratio, large nuclei, well-defined nucleus and pleomorphism. Highly mitotic activity with atypical mitosis and granular eosinophilic cytoplasm was also observed (figure 2). In addition, immunohistochemical (IHC) staining revealed that the samples were positive for CK5/6 and P63 markers, sensitive *markers* for squamous differentiation, which had confirmed our diagnosis (Figure 3). The patient was diagnosed as a case of poorly differentiated (basaloid) squamous cell carcinoma of pancreas, a rare condition, which arises from the pancreatic duct. All surgical margins including bile duct/choledochal surgical were free of tumor invasion. Although, 2 out of 6 resected lymph nodes were involved by tumor invasion and evidence of vascular invasion was seen, the invasion of the superior mesenteric artery (SMA) could not be assessed. The staging was confirmed as stage 2b (T3N1M0). Extensive workup was performed to evaluate other primary sources and metastatic disease did not indicate any head and neck malignancy. 

The patient tolerated the chemotherapy regimen well except a mild nausea and vomiting episode which was managed by 80 mg granisetron intravenously (IV). Vital signs, liver and renal functions were normal, and the patient was hemodynamically stable. The Eastern Cooperative Oncology Group (ECOG) performance status of patient was 1. The oncologist considered the adjuvant chemotherapy with a combination of 5-fluorouracil (5-FU) continuously infused over 120 h at a dose of 800 mg/m^2^/day in normal saline 1 liter on D1 through D5. Cisplatin (60 mg/m^2^) infused for 2h on D1. The treatment protocol was repeated every 3 weeks. After 3 chemotherapy cycles, the oncologist repeated the CT scan to evaluate the chemotherapy tumor’s response in the patient. The CT scan revealed no evidence of tumor’s shrinkage and there was a new metastatic lesion in the liver measured up to 40 mm. Furthermore, the patient’s chemotherapy regimen was change to 8 courses of FOLFIRI regimen (including irinotecan 180 mg/m^2 ^IV on day 1 and leucovorin 400 mg/m^2^ over 2 h IV on D1 followed by 5-FU 400 mg/m^2 ^IV bolus on day 1, then 5-FU 2400 mg/m^2^ as a 46-h continuous infusion. The treatment protocol was repeated every 2 weeks. 

After 8 course FOLFIRI chemotherapy completion, again the patient underwent the follow-up CT scan showed increase in size of liver metastasis and appearance of new pulmonary nodules lesion in the patient’s lung. The disease was considered progressive one. As the third line treatment, the patient received 125 mg/m^2^ protein-bound paclitaxel, nab-paclitaxel, IV followed by 1 g/m^2^ gemcitabine IV on days 1,8,15 repeated every 28 days. The treatment was considered to continue until unacceptable toxicity or evidence of disease progression. Premedication consisted of standard antiemetic drugs. After 2 cycles of chemotherapy, follow-up CT scan revealed that the patient’s condition was stable. The patient had less than one-month progression-free disease (PFS) time and he finally died after 13 months from tumor’s diagnosis time (Overall survival (OS): approximately 13 months).

## Discussion

Squamous cell carcinoma of the pancreas is a rare condition, responsible for 0.5 to 2% of all exocrine pancreatic tumors, and has not been well described since the 1940s. There are several hypothesis for identifying the origin of the tumor: 1- capability of malignant changing of bipotential primitive cell that is differentiated into either squamous or glandular carcinoma; 2- malignant transformation of squamous metaplasia of the ductal epithelium; 3-preexisting adenocarcinoma that transforms into SCC; 4-a transformation of atypical squamous cell into malignant cell (4, 5).The SCC clinical presentation of pancreas is similar to adenocarcinoma. Anorexia, weight loss, abdominal pain, nausea, vomiting, and obstructive jaundice are frequent complaints in patients present with pancreatic malignancy (6, 7).

The diagnosis of SCC of pancreas is a matter of exclusion and must be made after excluding metastases from another possible primary SCC (8). To establish a diagnosis of SCC in the pancreas, tumor enhancement on contrast CT, and tumor blush patterns on angiography, endoscopic retrograde cholangiopancreatography, and positron emission tomography-computed tomography scan (PET-CT scan) can be helpful (9). Recently, there has been a consensus on utilizing endosonography-guided fine needle aspiration (EUS-FNA) for diagnosing of SCC of the pancreas (10). Zhang et al. in 2018 reported a case of SCC of pancreas who was diagnosed by EUS-FNA. They also performed 18F-fluorodeoxyglucose (18F-FDG) PET/CT examination showed high 18F-FDG accumulation in pancreas for diagnosis and for the first time follow-up (10). The other similar reports also applied PET/CT examination for cancer detection (3). PET/CT has been noted to be a highly sensitive and accurate method for detecting pancreatic cancer. The reported sensitivity ranges from 78% to 95%, and accuracy from 64% to 91% (11). From past time, CT scans are often used to diagnose pancreatic cancer clearly and even have the ability to detect affected lymph nodes and near or distant organs. Furthermore, the cancer diagnosis of our patients was based on whole body CT examination (12). Off note, performing PET/CT is an inaccessible and expensive procedure and it limits to more complicated cases for diagnosis in our country. The rarity of SCC of pancreas made difficulties in meticulous diagnosis in clinical setting. Although, the data reported from the histopathological examination of our patient were in contrary with previous studies with cloud of squamous cells that had a positive response to P63 and CK5/6 markers staining. Adenosquamous carcinoma, another rare exocrine pancreatic neoplasm, should be usually considered in the differential diagnosis of SCC in which at least 30% malignant squamous cell carcinoma mixed with ductal adenocarcinoma (13). No evidence of ductal adenocarcinoma was seen in the histopathological examination of our patient’s sample. 

As it was performed in our patient, curative resection of tumor is highly recommended in pancreatic cancer to enhance the patient’s median OS in operable condition. Previous reports have noted 7-month median OS in 8 patients with pancreatic SCC undergoing curative surgical resection (14, 15). Brown et al. (28) indicated a median OS of 7 months (range 616 months) for patients who underwent curative resection and a recently published systematic review and pooled survival analysis by NtanasisStathopoulos et al. demonstrated a median OS of 7 months in 54 patients with pancreatic SCC. This report is the largest pool data analysis in the term of pancreatic SCC till now (16). 

They showed that operable cases had significantly better OS when compared with nonoperable cases (10 months versus 4 months, respectively) (3). Also, in this pool analysis, the patients’ median age at diagnosis was 63 years (range 33-80 years); the majority (61.1%) of cases were males; abdominal pain (77.8%) and weight loss (57.4%) were the most common chief complaints. Most of the patients were nonsmokers (77.8%) and non-alcoholic (70.4%). Most of the tumors were located in head of the pancreas (52.9%) followed by the tail (21.6%) and body (5.9%). The clinical feature and demographic characteristics of our patient was in contrary with other cases analyzed in pool data analyses. In our report, 13 months OS was seen from the patient’s diagnosis until the patient’ death which was one of the largest survival times ever been reported (17). 

Overall survival in patients who refuse treatment is dismal and has been reported as short as 3 months (3). However, there was no consensus on selecting the best therapeutic approaches. The cure rate for pancreatic cancer is very low at 7%, which makes the disease a real treatment challenge. Surgical resection is considered as the only potentially curative treatment and stage I, IIA and IIB pancreatic cancers are regarded resectable (3). Adjuvant radiotherapy and chemotherapy are usually accompanied with surgical therapeutic options. Among adjuvant chemotherapy regimen, the combination of cisplatin and 5-FU has shown promising results in terms of survival and the response rate in all types of pancreatic cancer and other SCC cancer originated from the other parts of body such as esophagus or head and neck. *Aurelllo et al. *reported more than 3-month OS with 2 cycles of cisplatin and 5-FU combination as adjuvant therapy in patients with pancreatic SCC (18). As a second-line treatment, the FOLFIRI regimen with manageable toxicity and notable efficacy has been suggested in platinum resistance pancreatic cancer with up to 6.6 months’ OS and 3.2-month PFS (8). The selection of chemotherapy regimens in our case was based on previous suggested chemotherapy and the oncologist preference and experiences. Combination of cisplatin and 5-FU as a first option and FOLFIRI regimen as an alternative were considered in the treatment of our case. 

In Frank et al.’s study, maintenance FOLFIRI regimen after induction with FOLFIRINOX resulted in 11 months PFS and 46 months OS (17). Moreover, FOLFIRI has demonstrated efficacy in squamous cell carcinoma of esophagus (19). However, our patient clinical response was not acceptable by administering the suggestive alternative chemotherapy regimen FOFIRI. 

According to several reports, gemcitabine-based chemotherapy including gemcitabine and carboplatin, oxaliplatin, 5-FU, and paclitaxel has been recommended as one of the therapeutic options in SCC pancreatic cancer (3, 15, 20). In addition, the new pharmaceutical form of paclitaxel such as nanoparticle albumin-bound paclitaxel or albumin‑free nanosomal paclitaxel lipid suspension in combination with gemcitabine has demonstrated significant efficacy in the treatment of pancreas cancer (3). By considering the new recommendation, we chose the combination of gemcitabine and nab-paclitaxel as the third-line treatment in our patients that led to stabilize disease progression and to the increase of OS of patients up to 13 months. Majumdar et al. administered nanosomal paclitaxel suspension in combination with gemcitabine for patients with SCC of the pancreas that resulted in more than 1-year OS while it had manageable toxicity (3). According to these reports, a newer pharmaceutical dosage form of paclitaxel and gemcitabine could be considered as a feasible and safe therapeutic option for patients with SCC of the pancreas. 

Other reports in regard with the use of new emerging monoclonal antibodies such as cetuximab have failed to demonstrate a defensible efficacy in the treatment of another part of body SCC origin including head and neck cancer (21), skin cancer (23) and metastatic esophageal SCC. Even based on the published meta-analysis in 2020, not only did not cetuximab give rise to increasing survival of pancreatic SCC but also would exacerbate toxicity profile of adjuvant regimen (24). Furthermore, there is still the urging of requirement to develop the new chemotherapy regimens and introduce new therapeutic agents which have the potential to prolong the survival time of patients suffering from pancreatic SCC as a rare condition. 

In conclusion, SCC of pancreas is a rare condition with a poor prognosis. Up to now, there is no determined treatment available for pancreatic SCC. This case report shows that the combination of nab-paclitaxel formulation and gemcitabine can be a potential treatment option for the treatment of locally advanced SCC of pancreatic head after failure in the treatment with other chemotherapy regimens. Nevertheless, new treatment modalities are needed to improve the prognosis of patients with pancreatic SCC.
